# Interleukin-33 mediated regulation of microRNAs in human cord blood-derived mast cells: Implications for infection, immunity, and inflammation

**DOI:** 10.1371/journal.pone.0314446

**Published:** 2024-11-26

**Authors:** Sherin Bakhashab, Ghalya H. Banafea, Farid Ahmed, Nadia Bagatian, Ohoud Subhi, Hans-Juergen Schulten, Peter Natesan Pushparaj

**Affiliations:** 1 Department of Biochemistry, King Abdulaziz University, Jeddah, Saudi Arabia; 2 Institute of Genomic Medicine Sciences, King Abdulaziz University, Jeddah, Saudi Arabia; 3 Department of Medical Laboratory Technology, Faculty of Applied Medical Sciences, King Abdulaziz University, Jeddah, Saudi Arabia; 4 Department of Pharmacology, Center for Transdisciplinary Research, Saveetha Dental College and Hospitals, Saveetha Institute of Medical and Technical Sciences, Chennai, India; University of Verona, ITALY

## Abstract

Mast cell (MCs) activation is the driving force of immune responses in several inflammatory diseases, including asthma and allergies. MCs are immune cells found throughout the body and are equipped with numerous surface receptors that allow them to respond to external signals from parasites and bacteria as well as to intrinsic signals such as cytokines. Upon activation, MCs release various mediators and proteases that contribute to inflammation. This study aimed to identify microRNAs (miRNAs) that regulate MC response to interleukin-33 and their target genes using a model of human cord blood-derived mast cells (hCBMCs). hCBMCs were induced with 10 and 20 ng of recombinant human interleukin-33 (rhIL-33) for 6 and 24 h, respectively. Total RNA was extracted from these cells and miRNA profiling was performed using high-throughput microarrays. Differential expression of miRNAs and target analysis were performed using Transcriptome Analysis Console and Ingenuity Pathway Analysis. The most significant miRNAs in each condition were miR-6836-5p (fold change = 1.76, p = 3E-03), miR-6883-5p (fold change = -2.13, p = 7E-05), miR-1229-5p (fold change = 2.46, p = 8E-04), and miR-3613-5p (fold change = 66.7, p = 1E-06). Target analysis revealed that these miRNAs regulate mast cell responsiveness and degranulation by modulating the expression of surface receptors, adaptors, and signaling molecules in response to rhIL-33 stimulation. This study is the first miRNA profiling and target analysis of hCBMCs that will further enhance our understanding of the role of miRNAs in the immune response in a timely manner and their relevance for the development of a new therapeutic target for inflammatory disorders.

## Introduction

Mast cells (MCs) are a type of granulated immune cells found in most tissues. They play a crucial role in initiating inflammation by responding to various stimuli, including pathogens, such as bacteria and parasites, allergens, and endogenous signals [1]. MCs communicate with other cells by secreting and recognizing cytokines, such as interleukin-33 (IL-33), which are known for their ability to activate MCs. IL-33 belongs to the IL-1 family of cytokines and is expressed by both the structural and immune cells. It functions as an alarmin by activating MCs through interaction with the transmembrane receptor serum stimulation-2 (ST2; IL-1R4) expressed on the surface of MCs [2, 3]. This interaction stimulates MCs to release a variety of cytokines, chemokines, and growth factors [4], which are triggered by the intracellular signaling cascade activated by the interaction between IL-33 and ST2. This cascade promotes de novo synthesis and expression of a range of mediators by activating nuclear factor kappa B (NF-kB) and p38 mitogen-activated protein kinase (p38-MAPK) [5].

Approximately 30% of human genes are regulated by microRNAs (miRNAs) [6, 7], a class of short non-coding RNAs that was first discovered three decades ago. miRNAs are approximately 17–25 nucleotides in length and function by binding to their respective mRNA targets, thereby regulating their translation [8]. miRNAs play a significant role in the regulation of various cellular functions including proliferation, differentiation, and polarization [9]. Additionally, miRNAs regulate degranulation and cell-to-cell communication in MCs [10, 11]. While miRNA expression in MCs has been studied in response to IgE/antigen cross-linking of the high-affinity IgE receptor [11–13], there is a lack of research on miRNAs in human cord blood-derived MC (hCBMC) activated through other means. This study aimed to address this gap by utilizing a combination of miRNA and mRNA high-throughput microarrays to provide the first global miRNA-mRNA profile of hCBMCs in response to IL-33 stimulation. Furthermore, Ingenuity Pathway Analysis (IPA) was used to efficiently analyze target genes affected by the miRNA-mRNA regulatory network.

## Materials and methods

### Sample collection and ethical statement

Umbilical cord blood samples were collected from healthy donors without a family history of atopic disease or mast cell disorders after obtaining written informed consent from December 02, 2020, to September 04, 2021. This study was approved by the Biomedical Ethics Unit, Faculty of Medicine, KAU (Approval Number 590–20). Each sample consisted of cord blood pooled from three donors. CD34^+^ hematopoietic stem cells (HSCs) were isolated using Lymph prep (1.077 g/ml; Axis Shield, Oslo, Norway), followed by CD34 microbead labeling and magnetic activated cell sorting (Miltenyi Biotec Inc., Bergisch Gladbach, Germany).

### Human cord blood-derived mast cells (hCBMCs) culture and stimulation by IL-33

CD34^+^ HSCs were cultured in AIM-V medium (Thermo Fisher Scientific, Waltham, MA, USA) supplemented with recombinant human interleukin-6 (rhIL-6, 50 ng/mL, Thermo Fisher Scientific) and stem cell factor (rhSCF, 100ng/mL, Miltenyi Biotec Inc.) for 8–10 weeks to support their differentiation into hCBMCs. Phenotyping was accomplished using flow cytometry, cell imaging, and gene set enrichment analysis as previously reported [14]. hCBMCs were activated with either 10 or 20 ng/mL recombinant human IL-33 (rhIL-33; Sino Biological, Beijing, China) and incubated for 6 or 24 h to observe immediate and prolonged responses as previously described [15, 16].

### RNA isolation and microarray hybridization

Total RNA was isolated using the RNAeasy Mini Kit (Qiagen, Hilden, Germany), followed by On-column DNase digestion with the RNase-free DNase set (Qiagen) according to the manufacturer’s instructions. Two biological replicates for whole-transcript microarray experiments were performed using Affymetrix Gene Chip Human Gene 1.0 ST arrays (Thermo Fisher Scientific), according to the manufacturer’s instructions, as described previously [14, 17]. The arrays were washed and stained using GeneChip Fluidics Station 450 and FS450_007 Fluidics Profiles and scanned using an Affymetrix GeneChip® scanner 3000 7G.

### miRNA isolation and microarray hybridization

The miRNA was isolated using the miRNeasy Mini Kit (Qiagen, Hilden, Germany) followed by RNase-Free DNase Set (Qiagen, Hilden, Germany), and then hybridized on Affymetrix GeneChip miRNA 4.0 Array (Santa Clara, CA, USA) in two biological replicates. This type of miRNA array is capable of interrogating over 2,500 mature human miRNAs, which are present in miRBase Release 20.

This procedure was performed in accordance with the manufacturer’s instructions. A sample concentration of 650 ng/8 μL was used for each array. Polyadenylate tailing and biotin labeling were performed using the FlashTag Biotin HSR RNA Labeling Kit (Foster City, CA, USA). Biotin-labeled samples were mixed with a hybridization cocktail and prepared using the Applied Biosystems GeneChip Hybridization, Wash, and Stain Kit (Foster City, CA, USA). The mixture was applied to the array and hybridized in a GeneChip Hybridization Oven at 48°C and 60 rpm for 17± 1 h. Finally, the array was washed and stained using Fluidics Station 450, FS450_0002 module, and scanned using GeneChip Scanner 3000 7G, Affymetrix GeneChip Command Software (AGCC).

### Bioinformatic analysis and target prediction

Raw CEL files from hCBMCs mRNA and miRNA samples were subjected to quality control and analyzed using Transcriptome Analysis Console (TAC) software (Thermo Fisher Scientific) to generate differentially expressed gene (DEG) sets by comparing IL-33 induced hCBMCs with control (uninduced hCBMCs). Gene expression data were statistically analyzed using ANOVA and the variance was corrected with eBayes, which is important for small sample sizes. Log2 (fold change (FC)), and significance was defined as |FC| ≥ 2 for mRNA and |FC| ≥ 1.5 for miRNA with p < 0.05. The microarray data derived from this study were deposited in the Gene Expression Omnibus (GEO) database (www.ncbi.nlm.nih.gov/geo/) under accession numbers GSE224089 (mRNA) and 261489 (miRNA).

Target prediction was accomplished using IPA 9.0 (QIAGEN Silicon Valley, Redwood City, CA, USA) miRNA Target Filter feature, which predicts the targets by combining 8mer and 7mer sites complementary to the miRNA seed via TargetScan and searching Ingenuity® Knowledge Base, TarBase, and miRecords databases for any previously reported mRNA-miRNA interactions.

Annotations of the physiological and molecular functions of the mRNAs were accomplished using IPA’s Core Analysis feature. Figures displaying networks of miRNA and mRNA targets were created using Cytoscape 3.10.1 [18].

## Results

### miRNA profile of hCBMC’s response to IL-33

Among all four conditions, 45 differentially expressed miRNAs were identified, of which 21 were upregulated and 24 were downregulated, with a fold change greater than |1.5| and p < 0.05. Nine miRNAs were differentially expressed due to the induction of hCBMCs with 10 ng of IL-33 for 6 h, whereas 20 miRNAs were differentially expressed when the incubation was extended to 24 h (Table 1). Furthermore, 19 and 15 miRNAs were differentially expressed in response to induction with 20 ng IL-33 for 6 and 24 h, respectively (Table 2). The most significantly differentially expressed miRNA when hCBMCs were stimulated with 10 ng of rhIL-33 were: miR-6836-5p (accession number: MIMAT0027574; FC = 1.76; p < 0.0029) and miR-6883-5p (MIMAT0027666; FC = -2.13; p < 0.00007) for 6 and 24 hours, respectively. Similarly, the most significant miRNAs when the cells were stimulated with 20 ng rhIL-33, miR-1229-5p (MIMAT0022942; FC = 2.46; p < 0.0008) and miR-3613-5p (MIMAT0017990; FC = 66.7; p < 0.00000123) at 6 and 24 h, respectively. Among the 45 differentially expressed miRNAs, only the broadly expressed anti-inflammatory factor hsa-miR-146a-5p [19, 20] was upregulated in a time-dependent manner and shared by all four conditions, whereas the majority of miRNAs were not shared between two or more conditions (Table 3).

**Table 1:** Differentially expressed miRNAs in hCBMCs stimulated with 10 ng rhIL-33.

6 h			24 h		
miRNA	Fold Change	P-value	miRNA	Fold Change	P-value
**hsa-miR-6836-5p**	1.76	0.0029	**hsa-miR-6883-5p**	-2.13	0.00007
**hsa-miR-1236-5p**	1.52	0.0037	**hsa-miR-146a-5p**	7.09	0.0006
**hsa-miR-6883-5p**	-1.52	0.0041	**hsa-miR-4484**	6.14	0.0018
**hsa-miR-146a-5p**	4.08	0.0044	**hsa-miR-205-3p**	-1.52	0.0039
**hsa-miR-1185-1-3p**	1.68	0.016	**hsa-miR-6891-5p**	2.39	0.0079
**hsa-miR-3613-5p**	2.55	0.0272	**hsa-miR-6727-5p**	-1.58	0.0114
**hsa-miR-7845-5p**	1.57	0.0296	**hsa-miR-5187-5p**	-1.67	0.0131
**hsa-miR-149-5p**	1.62	0.041	**hsa-miR-1908-5p**	-6.24	0.0133
**hsa-miR-146b-3p**	3.14	0.0462	**hsa-miR-15b-5p**	-1.62	0.0134
**hsa-miR-3162-5p**	-1.56	0.0467	**hsa-miR-6765-5p**	-4.49	0.0174
**hsa-miR-7641**	1.62	0.0481	**hsa-miR-3135b**	-7.77	0.0222
			**hsa-miR-1343-5p**	-3.61	0.023
			**hsa-miR-4714-5p**	1.57	0.0312
			**hsa-miR-6503-3p**	-2.78	0.032
			**hsa-miR-6799-5p**	-1.53	0.0341
			**hsa-miR-4488**	-5.64	0.035
			**hsa-miR-4508**	-2.38	0.037
			**hsa-miR-2115-5p**	-7.46	0.0385
			**hsa-miR-4758-5p**	-1.95	0.0431
			**hsa-miR-3180-3p**	-1.87	0.0468

**Table 2:** Differentially expressed miRNAs in hCBMCs stimulated with 20 ng rhIL-33.

6 h			24 h		
miRNA	Fold Change	P-value	miRNA	Fold Change	P-value
**hsa-miR-1229-5p**	2.46	0.0008	**hsa-miR-3613-5p**	66.73	0.00000123
**hsa-miR-6891-5p**	3.53	0.0009	**hsa-miR-146a-5p**	10.27	0.0002
**hsa-miR-7114-5p**	-1.57	0.0013	**hsa-miR-7114-5p**	-1.68	0.0005
**hsa-miR-20b-3p**	1.54	0.0046	**hsa-miR-6883-5p**	-1.75	0.0006
**hsa-miR-1306-3p**	1.6	0.0052	**hsa-miR-196b-5p**	-1.55	0.0009
**hsa-miR-8082**	-1.51	0.0054	**hsa-miR-940**	1.75	0.0017
**hsa-miR-5187-5p**	-1.75	0.0084	**hsa-miR-4484**	3.95	0.0089
**hsa-miR-146a-5p**	3.37	0.0096	**hsa-miR-7843-3p**	1.64	0.0096
**hsa-miR-4484**	3.77	0.0106	**hsa-miR-5194**	3.04	0.0099
**hsa-miR-2115-5p**	-9.87	0.0223	**hsa-miR-5187-5p**	-1.61	0.0187
**hsa-miR-15b-5p**	-1.53	0.0239	**hsa-miR-6891-5p**	1.97	0.0264
**hsa-miR-503-5p**	-2.16	0.027	**hsa-miR-1229-5p**	1.6	0.0287
**hsa-miR-6503-3p**	-2.89	0.0277	**hsa-miR-1281**	2.16	0.0372
**hsa-miR-4485**	2.7	0.0296	**hsa-miR-155-5p**	13.36	0.0428
**hsa-miR-6833-5p**	-1.54	0.0393	**hsa-miR-3180-3p**	-1.86	0.0479
**hsa-miR-6799-5p**	-1.5	0.0407			
**hsa-miR-3180-3p**	-1.9	0.0424			
**hsa-miR-4443**	-5.9	0.0484			
**hsa-miR-6808-3p**	-4.43	0.0495			

**Table 3:** miRNA distribution among conditions.

Condition	Total	miRNA
10 ng rhIL-33, 24 h	1	hsa-miR-146a-5p
10 ng rhIL-33, 6 h		
20 ng rhIL-33, 24 h		
20 ng rhIL-33, 6 h		
10 ng rhIL-33, 24 h	1	hsa-miR-6883-5p
10 ng rhIL-33, 6 h		
20 ng rhIL-33, 24 h		
10 ng rhIL-33, 24 h	4	hsa-miR-6891-5p, hsa-miR-3180-3p
20 ng rhIL-33, 24 h		hsa-miR-5187-5p, hsa-miR-4484
20 ng rhIL-33, 6 h		
10 ng rhIL-33, 6 h	1	hsa-miR-3613-5p
20 ng rhIL-33, 24 h		
10 ng rhIL-33, 24 h	4	hsa-miR-6503-3p, hsa-miR-2115-5p
20 ng rhIL-33, 6 h		hsa-miR-6799-5p, hsa-miR-15b-5p
20 ng rhIL-33, 24 h	2	hsa-miR-7114-5p, hsa-miR-1229-5p
20 ng rhIL-33, 6 h		
10 ng rhIL-33, 6 h	8	hsa-miR-1236-5p, hsa-miR-3162-5p
		hsa-miR-7845-5p, hsa-miR-1185-1-3p
		hsa-miR-149-5p, hsa-miR-146b-3p
		hsa-miR-7641, hsa-miR-6836-5p
10 ng rhIL-33, 24 h	10	hsa-miR-1908-5p, hsa-miR-205-3p
		hsa-miR-6765-5p, hsa-miR-4714-5p
		hsa-miR-4508, hsa-miR-1343-5p
		hsa-miR-3135b, hsa-miR-6727-5p
		hsa-miR-4488 hsa-miR-4758-5p
20 ng rhIL-33, 6 h	8	hsa-miR-8082, hsa-miR-20b-3p
		hsa-miR-6833-5p, hsa-miR-1306-3p
		hsa-miR-4485, hsa-miR-6808-3p
		hsa-miR-503-5p, hsa-miR-4443
20 ng rhIL-33, 24 h	6	hsa-miR-196b-5p, hsa-miR-155-5p
		hsa-miR-940, hsa-miR-7843-3p
		hsa-miR-5194, hsa-miR-1281

### Target analysis for miR-6836-5p

**Fig 1:**
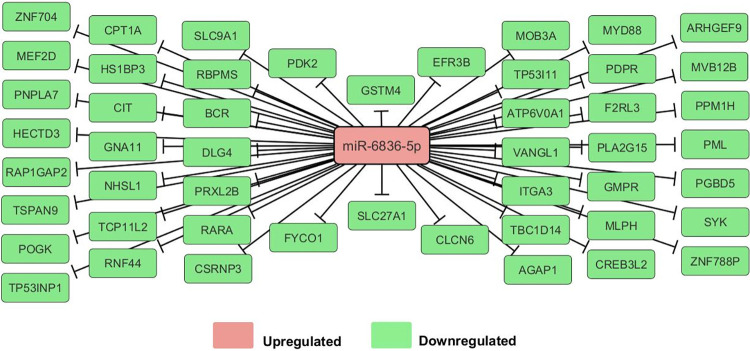
Network of genes targeted by miR-6836-5p. IPA identified 49 genes that were down-regulated in response to increased miR-6836-5p expression.

A total of 83 transcripts were predicted by IPA to be targeted by miR-6836-5p upon induction with 10 ng of IL-33 for 6 h. The transcriptomic profile of rhIL-33 stimulated hCBMCs confirmed that 49 of these transcripts were down-regulated (Fig 1). The mRNAs were classified using IPA core analysis, which revealed that the most enriched physiological functions were embryonic, respiratory, and hematological system development and functions, as assessed by Fisher’s exact test (P < 0.05). The top molecular functions were cell death and survival, post-translational modification, and protein folding, as determined using the same method (P < 0.05).

### Target analysis for miR-6883-5p

**Fig 2:**
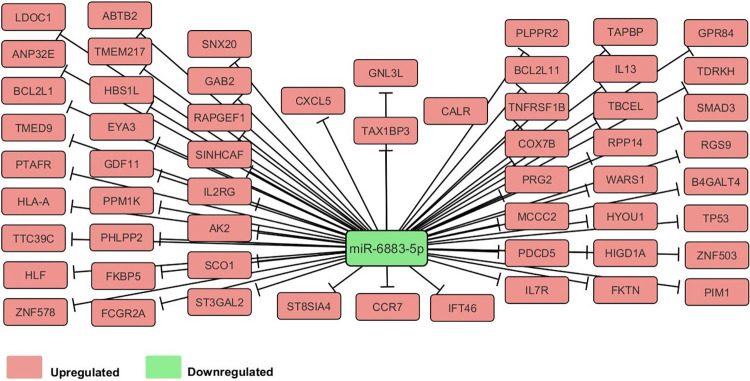
Network of genes targeted by miR-6883-5p. IPA identified 57 genes that were up-regulated in response to decreased miR-6883-5p expression.

A decrease in miR-6883-5p levels was observed in response to rhIL-33 stimulation, and it was predicted that approximately 104 mRNA targets were regulated by the miRNA upon induction with 10 ng of IL-33 for 24 h. Among these genes, 57 were upregulated in response to a decrease in miR-6883 expression (Fig 2). The top three physiological functions (p < 0.05) were hematological system development and function, immune cell trafficking, and lymphoid tissue structure and development. Cell signaling, cell-to-cell signaling and interaction, and cellular development were the top three molecular functions (P < 0.05).

### Target analysis for miR-1229-5p

**Fig 3:**
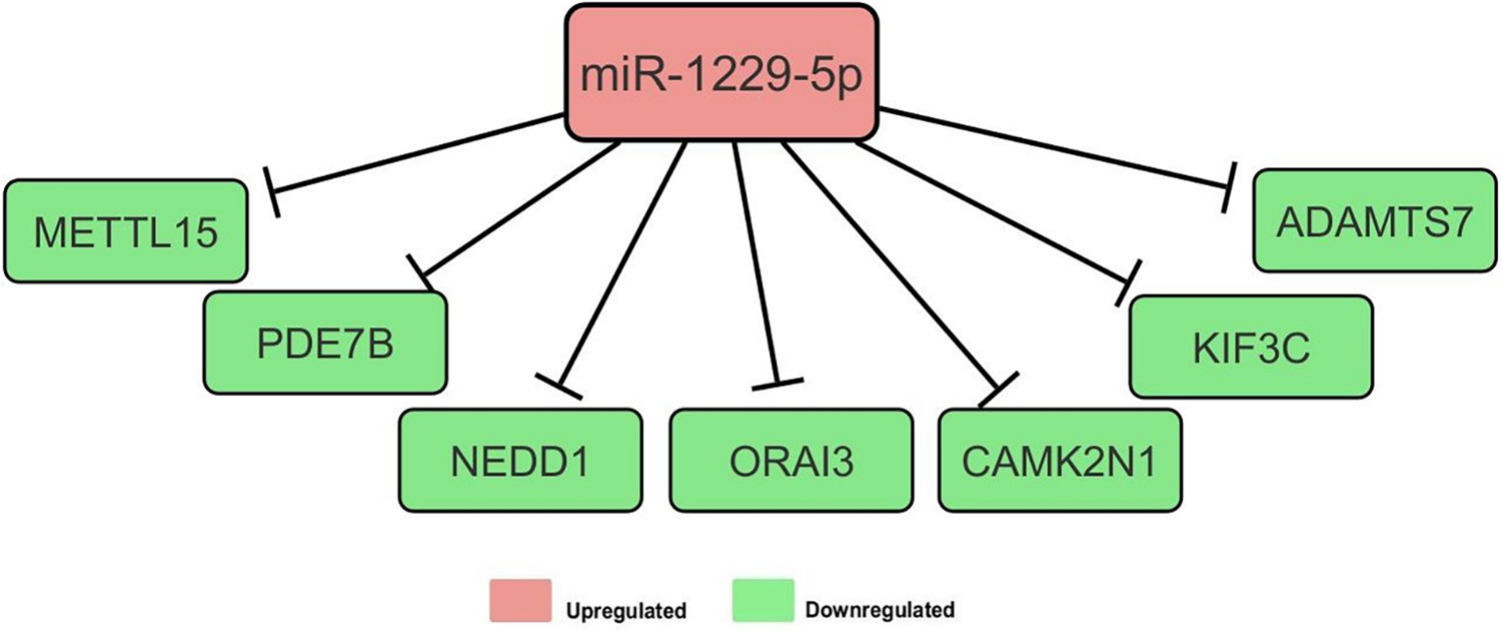
Network of genes targeted by miR-1229-5p. IPA identified seven genes that were downregulated in response to increased miR-1229-5p expression.

The induction of hCBMCs by 20 ng IL-33 for 6 h resulted in a significant upregulation of miR-1229-5p expression. Under these conditions, 19 mRNAs were predicted to be regulated by miR-1229-5p, of which 7 were downregulated (Fig 3). The physiological functions (p < 0.05) augmented by these targets included hematological system development and function, immune cell trafficking, and digestive system development and function. The molecular functions (P < 0.05) driven by these targets included cell morphology, cell-to-cell signaling and interaction, and carbohydrate metabolism.

### Target analysis for miR-3613-5p

**Fig 4:**
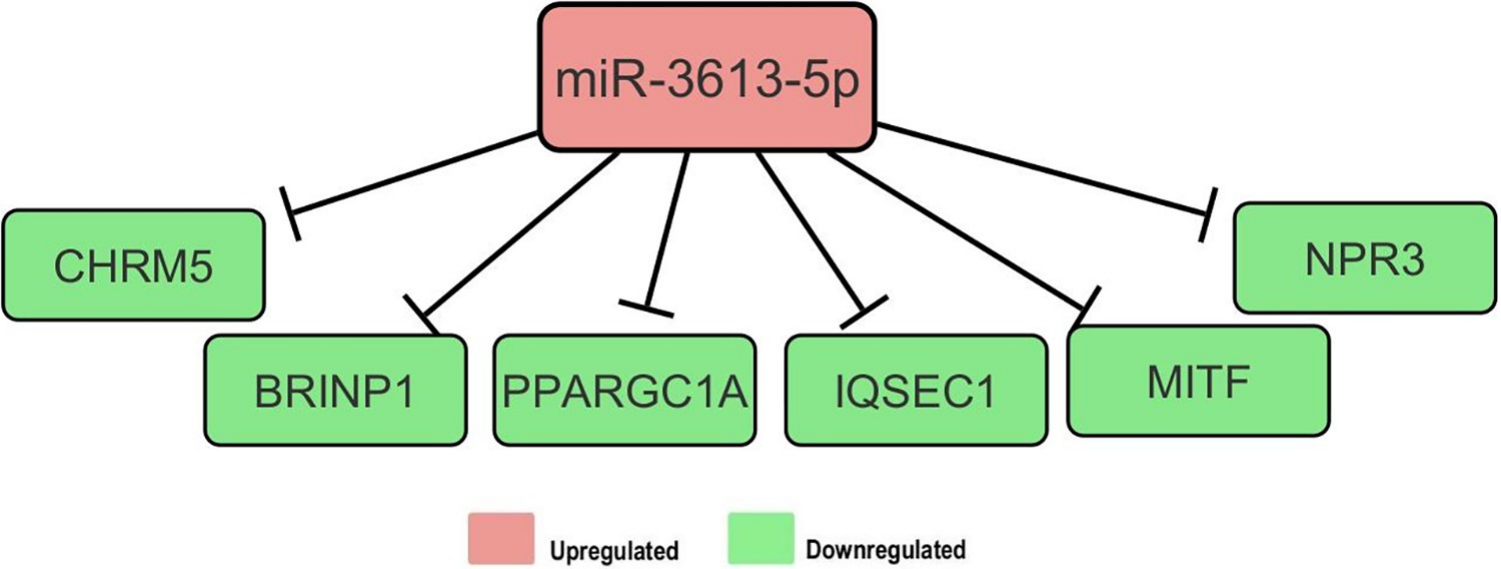
Network of genes targeted by miR-3613-5p. IPA identified six genes that were downregulated in response to increased miR-3613-5p expression.

miR-3613-5p was the most significantly differentially expressed miRNA when hCBMCs were induced with 20 ng of IL-33 for 24 h. This miRNA was predicted to regulate 17 genes using IPA software, of which six were experimentally confirmed to be downregulated (Fig 4). The enriched physiological functions (P < 0.05) included the cardiovascular system, connective tissue, hair, skin development and functions, and molecular functions (P < 0.05), such as cellular development, cell growth and function, and cell cycle.

## Discussion

Previous studies have characterized the miRNA profiles of MCs activated by antigen-IgE crosslinking [11–13]. However, the role of miRNAs in orchestrating the inflammatory response of human MCs remains unclear. Therefore, this study analyzed the global miRNA expression in hCBMCs under inflammatory conditions stimulated by IL-33 induction.

The data revealed 45 differentially expressed miRNAs in the hCBMCs in response to IL-33 treatment. Focusing on the most statistically significant miRNAs in each condition, we performed a target analysis and filtered the predicted targets by utilizing the gene expression profiles of IL-33 induced MCs; thus limiting the predicted targets to differentially expressed genes and allowing the identification of functions governed by miR-6836-5p, miR-6883-5p, miR-1229-5p, and miR-3613-5p. Target analysis suggested that the four miRNAs fine-tune inflammation by regulating the proliferation, differentiation, and activation of MCs, as well as intercellular communication.

IPA core analysis associating miR-6836-5p with tissue development, cell death, and survival. Łuczkowska and colleagues observed an increase in the miRNA’s level in cells treated with Bortezomib, an anti-cancer drug that promotes apoptosis [21], whereas it was decreased in samples from patients suffering osteonecrosis [22]. Moreover, miRNAs inhibit transcription factor ap-2 beta (*TFAP2B*), a transcription factor that increases cell proliferation and prevents terminal differentiation [21], which is consistent with our core analysis. Target analysis of miR-6836-5p suggested another role in regulating MC function by repressing MYD88, an adapter protein required for IL-33/ST2 signaling, which is essential for MC proliferation and production of IL-6 and IL-13 in response to IL-33 [23–25]. Although MCs respond to IL-33 by releasing various mediators, they do not evoke degranulation after short-term exposure [26]. miR-6836-5p was predicted to target RARA, SYK, and BCR, which facilitate IgE crosslinking, calcium mobilization, and cytoskeleton rearrangement required for MC degranulation, suggesting a possible mechanism by which miR-6836-5p downregulates MC degranulation [27–29]. Moreover, ITGA3, an integrin crucial for the adhesion and migration of MCs in the lungs and blood vessels, is targeted by miR-6836-5p, suggesting that this miRNA regulates MCs mobilization [30].

**Fig 5:**
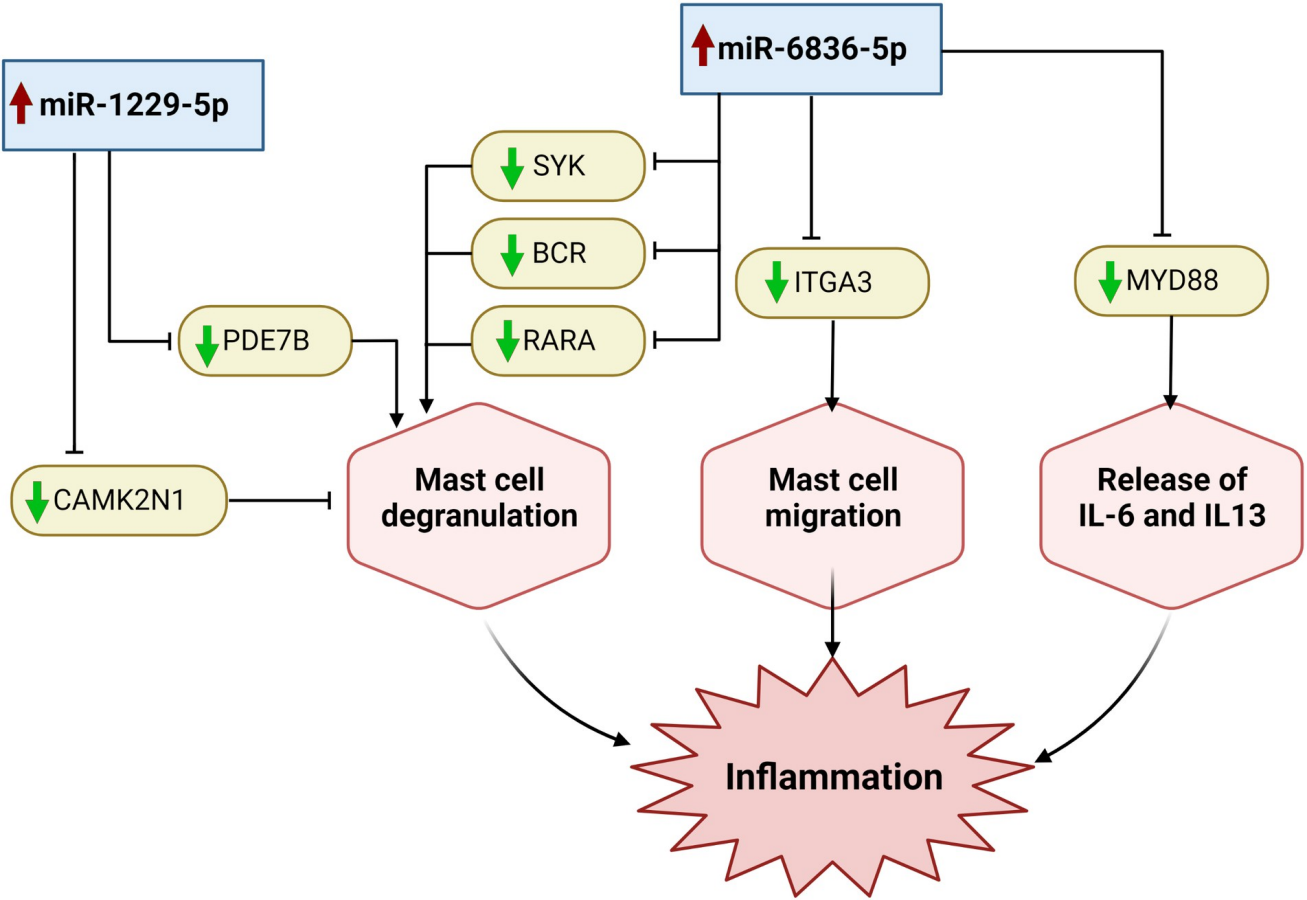
miRNA regulation of mast cell function during acute inflammation. When hCBMCs were stimulated with IL-33 for 6 h, the most significantly differentially expressed miRNAs were miR-6836-5p and miR-1229-5p, which in turn regulate the genes involved in mast cell functions that contribute to inflammation. Key: Upward red arrow: upregulation; downward green arrow: downregulation; → promotion; ⊣: inhibition.

Subjecting hCBMCs to a higher concentration of IL-33 (20ng/mL) for 6 h upregulated the expression of miR-1229-5p which has been reported to downregulate MAPK1, an enzyme with an integral role in rearranging the cytoskeleton of MCs to facilitate degranulation [31–33]; aligning with IPA functional analysis of miR-1229-5p which revealed that cell morphology was among the top significant functions regulated by this miRNA. Intriguingly, target analysis revealed that the miRNA targeted two transcripts with contradictory effects on degranulation, PDE7B and CAMK2N1. PDE7B supports MC proliferation and degranulation by lowering the intracellular cAMP concentration [34–36], whereas IL-33 activates calcium-calmodulin-dependent kinase II (CAMK2) by downregulating its inhibitor, CAMK2N1, thus inhibiting autophagy and preventing degranulation [37–39]. The roles of miR-6836-5p and miR-1229-5p in regulating the acute response of MCs to IL-33 are summarized in (Fig 5).

In contrast to the other miRNAs, miR-6883-5p was downregulated in response to IL-33 after 24 h of exposure. miRNAs are transcribed from the intronic region of the tumor suppressor gene Period Circadian Regulator 1 (*PER1*) [40]. Recent advances in circadian rhythm research have uncovered a synchronized connection between MC-specific genes encoding FcεRIα, CD117, MC proteases, and circadian clock genes including *PER1* [41]. Previous studies have also revealed that upregulation of miR-6883-5p regulates proliferation by inducing cell cycle arrest [40, 42]. IPA functional analysis revealed its involvement in the regulation of cell signaling. Starting with cell surface receptors, miR-6883-5p was shown to target CCR7, IL7R, TNFRSF1B, IL2RG, FCGR2A, and GAB2, which are chaperone-binding proteins required for the assembly of major histocompatibility complex I and MC antigen-presenting functions [43, 44]. Furthermore, this miRNA has been shown to target CALR and PIM1, which are involved in MC differentiation and recruitment, respectively [45–47], as well as IL-13, which exacerbates the hyper-responsiveness and airway obstruction caused by MCs in asthma patients [48], all of which are upregulated in MC’s in response to IL-33 and a decrease in miR-6883-5p.

Other targets of miR-6883-5p include the protein phosphatase encoded by *BCL2L1*, which prevents the activation of pro-inflammatory cytokines IL-1β, IL-18, and IL-33 [49]; the transcription factor *SMAD3*, which reduces the production of IL-6 and TNF-α, thus downregulating the MC-mediated innate immune response [50]; and *TP53*, which reduces MC activation by inhibiting the NF-κB pathway [51]. This miRNA also targets BCL2L11, also known as BIM, a pro-apoptotic member of the BCL2 family [52].

The expression of miR-6883-5p decreased in response to prolonged exposure to IL-33, allowing for the upregulation of its targets. The gene sets regulated by this particular miRNA possess opposing effects, as the first set of receptors exhibits proinflammatory effects, whereas the second set of transcription factors exhibits anti-inflammatory effects; however, this paradox is a common phenomenon of miRNAs [53, 54].

After 24 h of stimulation with 20ng/mL IL-33, expression miR-3613-5p increased six-fold. Among the four differentially expressed miRNAs, | FC| was the highest. TarBase (V. 8) registered 212 experimentally validated miRNA targets. miR-3613-5p was identified as a tumor repressor in pancreatic cancer cells by targeting cyclin-dependent kinase 6 (CDK6), thus regulating the cell cycle [55]. However, it has also been reported to promote the proliferation of lung adenocarcinoma cells through indirect activation of MAPK signaling [56].

Additionally, miR-3613-5p was found to be decreased in plasma sample ocases of graft versus host disease, where it was predicted to target several genes involved in regulating immune response signaling [57]. IPA revealed that miR-3613-5p was mainly involved in cellular development and allergies. miRNAs target two major transcription factors: PPARGC1A, which enhances MC viability [58], and MITF, which is essential for MC differentiation, development, and function [33, 59].

**Fig 6:**
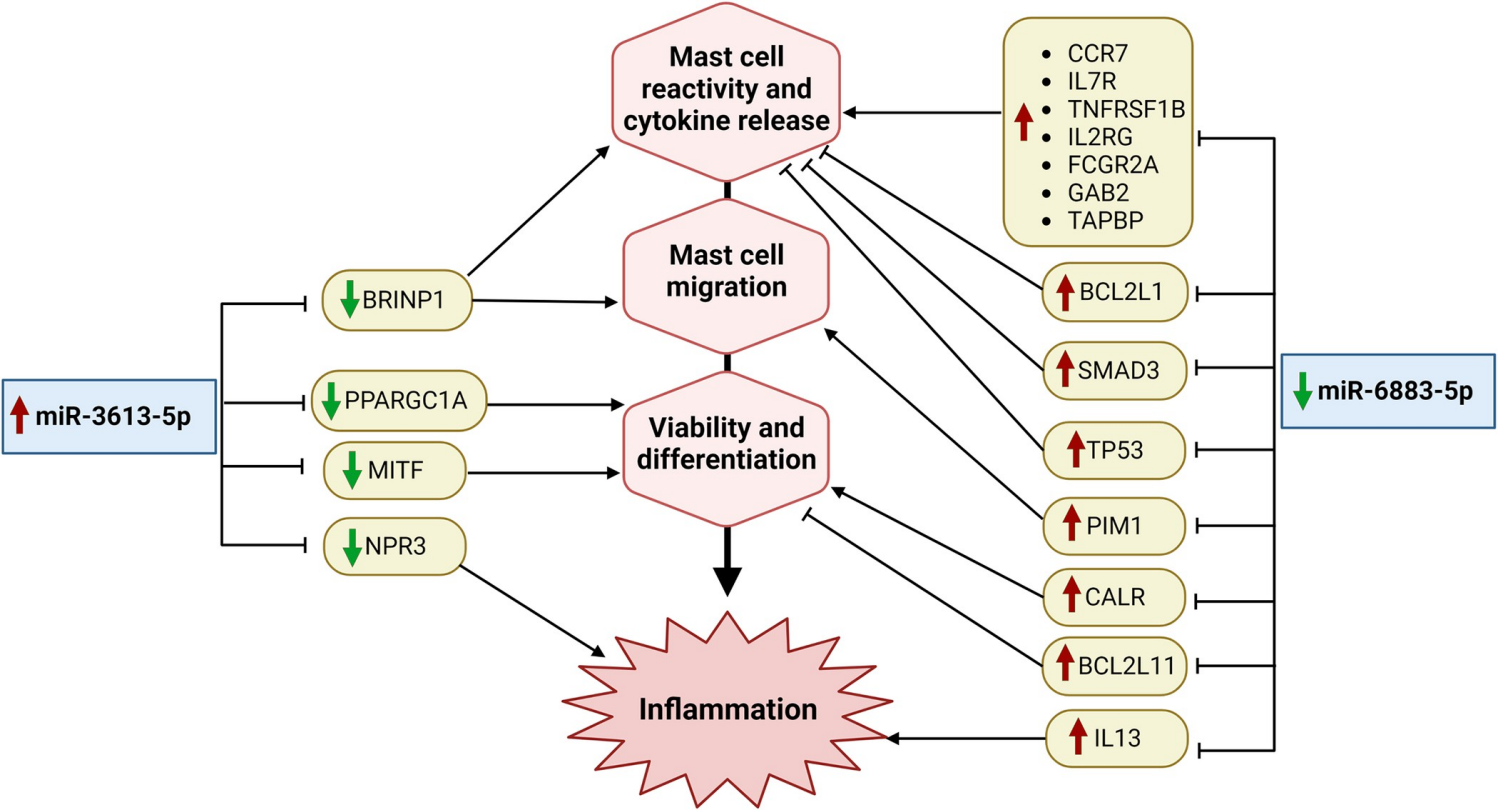
miRNA regulation of mast cell function during prolonged inflammation. When hCBMCs were stimulated with IL-33 for 24 h, the most significantly differentially expressed miRNAs were miR-6883-5p and miR-3613-5p, which in turn regulated numerous genes involved in mast cell functions that contribute to inflammation. Key: Upward red arrow: upregulation; downward green arrow: downregulation; → promotion; ⊣: inhibition.

Moreover, miR-3613-5p targets NPR3, a receptor that facilitates internalization, clearance, and degradation of anti-inflammatory natriuretic peptides [60, 61]. Another target is BRINP1, which belongs to the family with sequence similarity 5 (FAM5) and is homologous to BRINP3, which increases NF-κB activation and expression of adhesion molecules during inflammation [62]. Thus, the miR-3613-5p increase after 24 h leads to the downregulation of hCBMCs viability. The roles of miR-6883-5p and miR-3613-5p in regulating the prolonged response of MCs to IL-33 are summarized in (Fig 6).

Most differentially expressed miRNAs were not shared between conditions; however, miR-146a-5p expression increased under all conditions in a time-dependent manner. miRNAs are known for their anti-inflammatory effects and can be found in exosomes released from human MC cell lines stimulated by calcium ionophores [10] and exosomes from mouse MCs activated by IL-33 [63]. The changes in miRNAs in immune cells during their response to IL-33 remain poorly understood. Chia and colleagues have studied the differential miRNA expression in rat macrophages stimulated by IL-33, however the only mutually differentially expressed miRNA with this project was the proinflammatory miR-155-5p [64].

### Therapeutic prospects

IL-33 plays a crucial role in activating MCs and in enhancing the immune response against mechanical injury and bacterial and parasitic infections. However, IL-33 driven inflammation is a double-edged sword capable of prolonging inflammation and inducing tissue damage, as portrayed in asthma and allergy, and precise modulation is essential to sustain homeostasis, and herein lies the potential therapeutic role of miRNAs.

Targeting miRNAs has promising therapeutic applications because of their ability to post-transcriptionally regulate gene expression. In our model, we identified miRNAs that fine-tuned MC response to IL-33 driven inflammation and their respective targets. miRNAs are linked to physiological and molecular functions that are hallmarks of inflammation. For example, miR-6883-5p, which is downregulated in response to IL-33, targets genes involved in the responsiveness, proliferation, and recruitment of MCs to sites of inflammation. Using mimics of miR-6883-5p may be a promising approach to control the progression of MC-driven inflammation; however, to achieve the potential of miRNAs as therapeutic agents, several challenges must be overcome, such as the miRNA delivery method and the avoidance of off-target effects.

### Limitations and future directions

Isolating mature MCs presents a challenge due to the low numbers of human primary MCs obtained as tissue-resident immune cells for cellular and molecular studies. This is because they comprise only a small percentage of cells in the tissues. Additionally, MCs exhibit a range of phenotypes and perform multiple functions during homeostasis and various disease states. These functions are influenced by the MCs’ residence in tissues and the stimuli they receive in specific tissue microenvironments. The small sample size in this study was attributed to donor dependency, which plays an important role in the variability in the expression levels of hCBMC-specific markers. Therefore, considering various donors as possible providers of CD34+ HSCs can yield more reliable results, which consequently leads to higher costs [65]. The variation in hCBMC-specific markers can only be detected at the end of cell differentiation at eight weeks.

Another limitation of this *in vitro* study lies in the utilization of microarray technology, which, although it is a high-throughput and robust technique and depends on the design of the microarray, can only analyze a restricted number of miRNAs and fails to identify novel miRNA sequences. Furthermore, microarrays can exhibit difficulties in differentiating similar sequences owing to suboptimal probe-miRNA binding, which may lead to false positives. The findings of this study serve as a guide for future investigations, and the expression of miRNAs and their impact on their respective targets should be examined in functional in vivo studies to gain a more comprehensive understanding of miRNA regulation of IL-33 effects on MCs.

## Conclusion

In this study, we unveiled previously unknown roles of miR-6883-5p, miR-6836-5p, miR-1229-5p, and miR-3613-5p in modulating the response to IL-33 induced inflammation in hCBMCs. miRNAs have been found to regulate key inflammatory processes, including MC proliferation, differentiation, and degranulation. These findings suggest that miR-6836-5p and miR-1229-5p regulate the acute phase of inflammation by controlling degranulation and intercellular communication, while miR-6883-5p and miR-3613-5p orchestrate the prolonged response, balancing the pro- and anti-inflammatory pathways. Through the integration of the transcriptome profiles of both miRNAs and mRNA, we identified their respective target genes such as SMAD3, TP53, and IL-13 and briefly characterized their inflammatory or anti-inflammatory functions during inflammation.

This comprehensive profile reveals the complicated regulatory network of miRNAs in mast cell-mediated inflammation and lays the groundwork for future research on therapies that target miRNAs to treat inflammatory disorders. Although our study offers novel insights, it is important to acknowledge its limitations, including the use of an in vitro model and microarray technology. To better understand how miRNAs control the effects of IL-33 on mast cells, further research should be conducted to confirm these results in functional in vivo studies. Despite these limitations, our study represents a significant advancement in understanding the intricate interplay between miRNAs and mast cell function in IL-33 the mediated immune response.
